# Leadership Practices, Organization Structure, and Other Factors Associated with Higher 2022–2023 US News and World Report Ranking

**DOI:** 10.1007/s11606-024-09028-7

**Published:** 2024-10-02

**Authors:** Divya Sankisa, Robert Tatum, Abhiraj Saxena, David J. Whellan, Rene J. Alvarez, Rohinton J. Morris, Vakhtang Tchantchaleishvili

**Affiliations:** 1 Sidney Kimmel Medical College, Thomas Jefferson University, Philadelphia, PA USA; 2 Department of Surgery, University of Vermont, Burlington, VT USA; 3 Division of Cardiology, Department of Medicine, Thomas Jefferson University, Philadelphia, PA USA; 4 Division of Cardiac Surgery, Department of Surgery, Thomas Jefferson University, Philadelphia, PA USA

## Abstract

**Background:**

*U.S.* *News* *and World Report* 2022–2023 hospital rankings were used to investigate the leadership practices and organizational structure of ranked healthcare institutions.

**Methods:**

Institutional variables including physician leadership status, executive board size, number of physician board members, type of physician practice, health system size, and for-profit status were collected. *US News and World Report* scores were used to create a comprehensive institutional rank order list. Strength of association was evaluated between these variables and the organization’s *US News and World Report* rank.

**Results:**

Our analysis included 546 institutions representing 1676 hospitals. Leadership under a physician CEO (OR 1.59 (95% CI 1.27, 2.00) *P* < 0.001), group practice (OR 1.25 (95% CI 1.01, 1.59) *P* = 0.042), and bed count of the institution’s highest-ranking hospital (OR 1.001 (95% CI 1.0009, 1.0014) *P* < 0.001) were associated with higher *US News and World Report* ranking. For-profit status was negatively associated with institution ranking (OR 0.44 (95% CI 0.24, 0.78) *P* = 0.005). No significant association was demonstrated between number of physician executive board members, health system bed count, executive board size, or the number of hospitals in a health system and the institutional ranking.

**Conclusions:**

Physician leadership, organizational structure, size of the institution’s flagship hospital, and tax status are significantly associated with an organization’s *US News and World Report* rank.

**Supplementary Information:**

The online version contains supplementary material available at 10.1007/s11606-024-09028-7.

## INTRODUCTION

Throughout the preceding decades, the healthcare business landscape has transformed into a dynamic marketplace in which organizations are characterized by cross-functional teams comprised of individuals with diverse backgrounds and widely varying professional competencies.^1^ Multidisciplinary transitions are accelerating away from the traditional fee-for-service healthcare spending model in favor of an outcomes-based compensation structure for hospital systems and physicians.^2^ The emergence of compensation packages that prioritize efficiency and penalize wasteful spending underscores the growing need to utilize value-based assessments to improve our healthcare institutions so we can optimize the care they provide and reduce overall costs.^3^ Pursuing these goals requires analyzing high-performing healthcare organizations to gain insight into the clinical factors and institutional management practices that underlie their success.

*US News and World Report* is an internationally recognized US-based multimedia organization that publishes what is widely considered the most influential hospital ranking system in the USA through *US News and World Report* Scores.^4^ Each year, *US News and World Report* evaluates more than 5000 hospitals and produces quality-based scores that inform patients making health-related decisions for themselves and their families. The yearly report assigns points to hospitals based on data acquired from the federal Centers for Medicare & Medicaid Services’ database, the American Hospital Association (AHA) database, and other professional organization data reports. *US News and World Report* evaluates hospital performance by utilizing metrics designed to assess quality across four broad categories: clinical outcomes, patient experience, care-related factors like provider staffing and breadth of institutional resources and services, and expert opinion.^5^

Previous studies investigating healthcare institution quality have focused on highly selected hospitals from the *US News and World Report Honor Roll*, the arbitrarily defined group of top-ranking healthcare organizations^ 6^^, ^^8^ The resulting narrow research focus is partially attributable to *US News and World Report* only providing this type of stratified analysis in their specialty-based rankings, leaving performance analyses of institutions not included in the *US News and World Report Honor Roll* largely unexamined. Although previous studies have found strong correlations between a range of administrative and clinical practices and hospital rankings ^9^^, ^^10^^, ^^11,^ missing from the literature is a comprehensive analysis that combines institutional structure, physician leadership status, and strength of crucial personnel and clinical resources to investigate the specific factors associated with higher rankings in the *US News and World Report* system. Identifying such factors and determining the impact each has on institutional performance requires understanding the basic architecture of rank lists and the criteria used to judge the organizations that *US News and World Report* evaluates.

A ranking system is a comparative model that assigns incremental superiority based on the score given to each entity within the rank—a useful and instructive framework that carries predictive value. This value is lessened, however, when top-tier performers are analyzed as an equal group and others entirely excluded. Furthermore, previous studies have predominantly analyzed hospitals individually and not as integral parts of the larger institutions they comprise.^12^ However, as the trend toward integration of hospitals into larger health systems accelerates, crucial organizational factors like executive leadership and governance practices will become increasingly influenced by decisions made at the health system level. This trend emphasizes the central and determinative role physician leadership plays within healthcare quality improvement metrics, employee satisfaction, and workplace hygiene factors that impact recruitment, retention, and overall engagement. Refining the corporate governance practices and optimizing the strategic decision-making processes within healthcare institutions that rely on competent physician leaders and motivated employees depend crucially on these clinical and administrative management teams defining mutual goals and creating tactics that prioritize shared visions.

The *US News and World Report* rankings are divided into specialty, conditions, and procedure rankings. Significantly more hospitals within each category are assigned scores than are ranked, and only the top 20–30 hospitals (“Honor Roll”) are listed in the overall rank. Our analysis included all available data from *US News and World Report* and AHA. The information on group practice was obtained from AHQR 2018 report. By analyzing the relationship between administrative characteristics and *US News and World Report* rank, we aspired to understand whether institution variables commonly perceived as favorable truly correlate with performance*.* Using this extensive rank order list, we sought to better understand the complex relationships between a hospital’s 2022–2023 *US News and World Report* ranking and the organizational structure and leadership practices at the health system level that may contribute to their success.

## METHODS

### Terminology

Throughout the manuscript, we use the term “institution” broadly to encompass both integrated health systems and independently governed standalone hospitals. The analysis is performed at the institutional level unless specified otherwise.

### Data Collection and Organization

Hospital-level data provided for each of the 15 clinical specialties listed on the official *U.S. News* website were extracted using a custom Python script. Next, we used hospital identifiers to calculate cumulative scores for each hospital by adding individual subspecialty scores for each 15 specialties. Hospitals ranked in fewer than three specialties were excluded from our analysis, and specialty ratings were excluded if data were available for fewer than 10% of listed hospitals. The implementation of numerical exclusion criteria based on information availability is an accepted method of establishing boundaries within data sets. The unrefined data set yielded 3154 hospitals; however, after we applied the 10% cutoff and three-specialty inclusion criteria, that hospital number decreased to 1676, a significantly more manageable data set. These restrictions provided focus and fostered project manageability without omitting potentially crucial research material. The following publicly available data sources were used to create a combined database: *U.S. News and World Report* (2022–2023), the American Hospital Association (AHA) (data from August 2022), and the Agency for Healthcare Research and Quality (AHRQ) (data from 2018).

*U.S. News and World Report* only provides discrete hospital rankings, so we used the AHA database to identify associations between individual hospitals and specific health systems. If a health system was provided, we used the top-ranked hospital within the system as a surrogate for the institution’s flagship (top-ranking) hospital. If no associated health system was provided by the AHA database or identified on the hospital’s website, then the hospital was included as a discrete entity. In this case, the standalone hospital was treated as a flagship hospital for statistical analysis.

Next, the AHA online database was used to identify hospital size, number of hospital beds, number of health system beds, for-profit status, executive board size, and chief executive officer status (including professional degree). If information was not available on the AHA database, then we attempted to collect data from publicly available online sources. Finally, we used data collected from the AHRQ to identify health systems considered group practices. For the purposes of this study, we used the AHRQ group practice definition which stated that a group practice was any medical practice that includes at least one hospital and is comprised of at least one group of physicians providing comprehensive care and are connected with each other and with the hospital through common ownership or joint management.^13^

### Statistical Analysis

Data were presented using standard descriptive methods including median and Interquartile range (IQR) for continuous variables and percentages for categorical variables. Institutional variables were entered as a priori predictors into the multivariable ordinal regression utilizing *US News and World Report* rank as the outcome variable. Our decision-making pertaining to multivariate and univariate analyses was informed by our preexisting variable limitations and scope of inquiry. Multivariable analysis of a priori selected variables is most useful when exploring the relationship between each variable and a specific outcome, since lack of association of certain variables with the outcome can be considered nearly as informative as the presence of a significant association. Given these factors, our strategy was to analyze all a priori variables at once in a multivariable analysis. Our variable selection framework was based on availability of relevant data. Pertinent variables that were either unavailable or unsuitable for systematic analysis were not included in the analysis. Ordinal regression results were presented as odds ratios (OR) with 95% confidence intervals, whereby higher ORs indicate higher institution ranking and vice versa. Odds ratios for continuous variables were reported “per-unit” change. Data points found to be associated with *US News and World Report* rank were further explored individually using Wilcoxon’s rank-sum test for categorical variables and Pearson’s correlation for continuous variables. Categorical variables were further examined with likelihood ratios (LR) by quartile. Conventional thresholds were used to define statistical significance (*P* < 0.05). R software, version 3.5.1 (R Foundation for Statistical Computing, Vienna, Austria), was used for data analysis and representation.

## RESULTS

### Characteristics of Analyzed Institutions

Four out of 15 specialties were excluded because they failed to meet the 10% threshold of hospital representation: Psychiatry, Otolaryngology, Ophthalmology, and Rheumatology. The remaining eleven specialties were included (Table 1). A total of 3153 hospitals were scored in any of these 11 specialties. Of these 1676 were scored in at least three specialties, representing 546 independent institutions (health systems, *n* = 429; standalone hospitals, *n* = 117) and were included in the analysis (Table 2). The top 20 institutions generated from this ranking were well aligned with the 2022–2023 *US News and Word Report Honor Roll* (Supplemental Tables 1 and 2).

**Table 1:** Number of Hospitals Scored by Number of Specialties by the *US News and World Report*

Specialty count	Cancer	CARD	ENDO	GASTRO	Geriatrics	NEURO	OBGYN	ORTHO	PULM	REHAB	URO	Total
11	77	77	77	77	77	77	77	77	77	77	77	77 (4.6%)
10	236	235	235	239	236	239	112	238	239	142	239	239 (14%)
9	242	221	209	253	243	251	27	251	252	76	252	253 (15%)
8	209	153	117	251	227	234	8	250	250	60	249	251 (15%)
7	87	85	52	204	177	174	3	201	204	52	196	205 (12%)
6	37	15	47	210	192	144	1	208	212	30	194	215 (13%)
5	9	3	19	192	189	73	1	195	200	26	143	210 (13%)
4	6	1	5	99	97	21	1	94	114	11	65	130 (7.8%)
3	3	1	0	43	61	14	0	50	71	7	38	96 (5.7%)
Total	906 (54%)	791 (47%)	761 (45%)	1568 (94%)	1499 (89%)	1233 (74%)	230 (14%)	1564 (93%)	1619 (97%)	481 (29%)	1453 (87%)	1676 (100%)

**Table 2:** Institutional Variables Analyzed

Variable	
Number of hospitals scored in any specialty	3153
*US News and World Report* score, median [IQR]	105.4 [0.0, 279.0]
Number of hospitals scored in at least 3 specialties	1676
*US News and World Report* score, median [IQR]	270.3 [187.2, 371.1]
Number of institutions (health systems or standalone hospitals) with a hospital scored in at least 3 specialties	546
*US News and World Report* score, median [IQR] Number of hospitals, median [IQR] Number of beds, median [IQR] Number of beds in the top-ranking hospital, median [IQR] Physician CEO, *n* (%) Number of physicians on executive board, median [IQR] Number of executive board members, median [IQR] Group practice For-profit	339 [229.8, 464.1] 3 [1, 7] 768 [305, 1596] 379 [234, 625] 118 (22%) 2 [1, 3] 11 [8, 15] 363 (66%) 28 (5.1%)
Number of health systems comprising more than one hospital Number of hospitals, median [IQR] Number of beds, median [IQR] Number of beds in the top-ranking hospital, median [IQR]	429 5 [3.0, 9.5] 1109 [609, 227] 426 [266, 672]
Number of standalone hospitals Number of beds, median [IQR]	117 241 [167, 330]

*IQR* interquartile range

### Leadership

**Figure 1:**
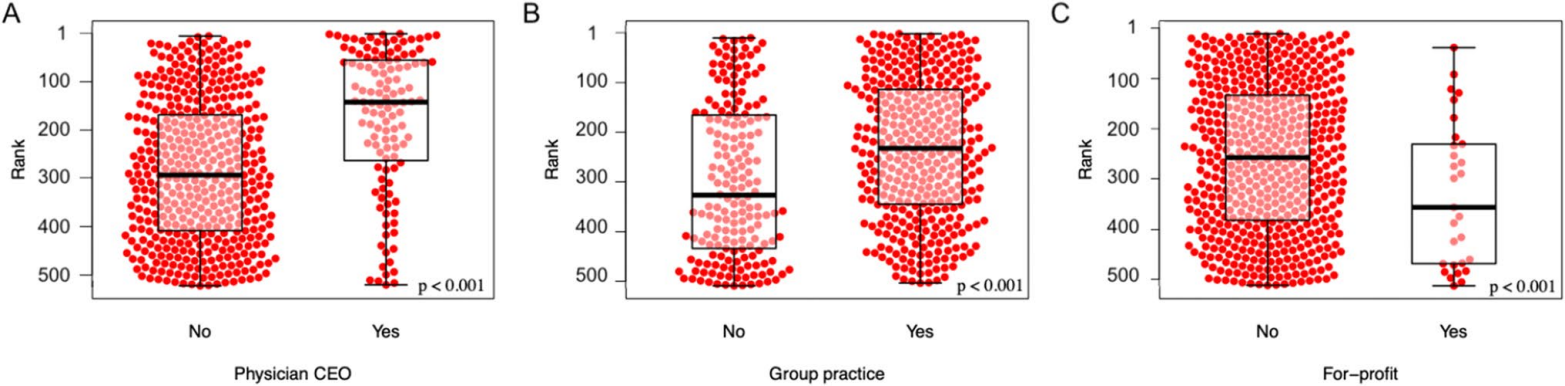
Comparative analysis of *US News and World Report* ranking by **A** physician CEO, **B** group practice, and **C** for-profit status.

**Figure 2:**
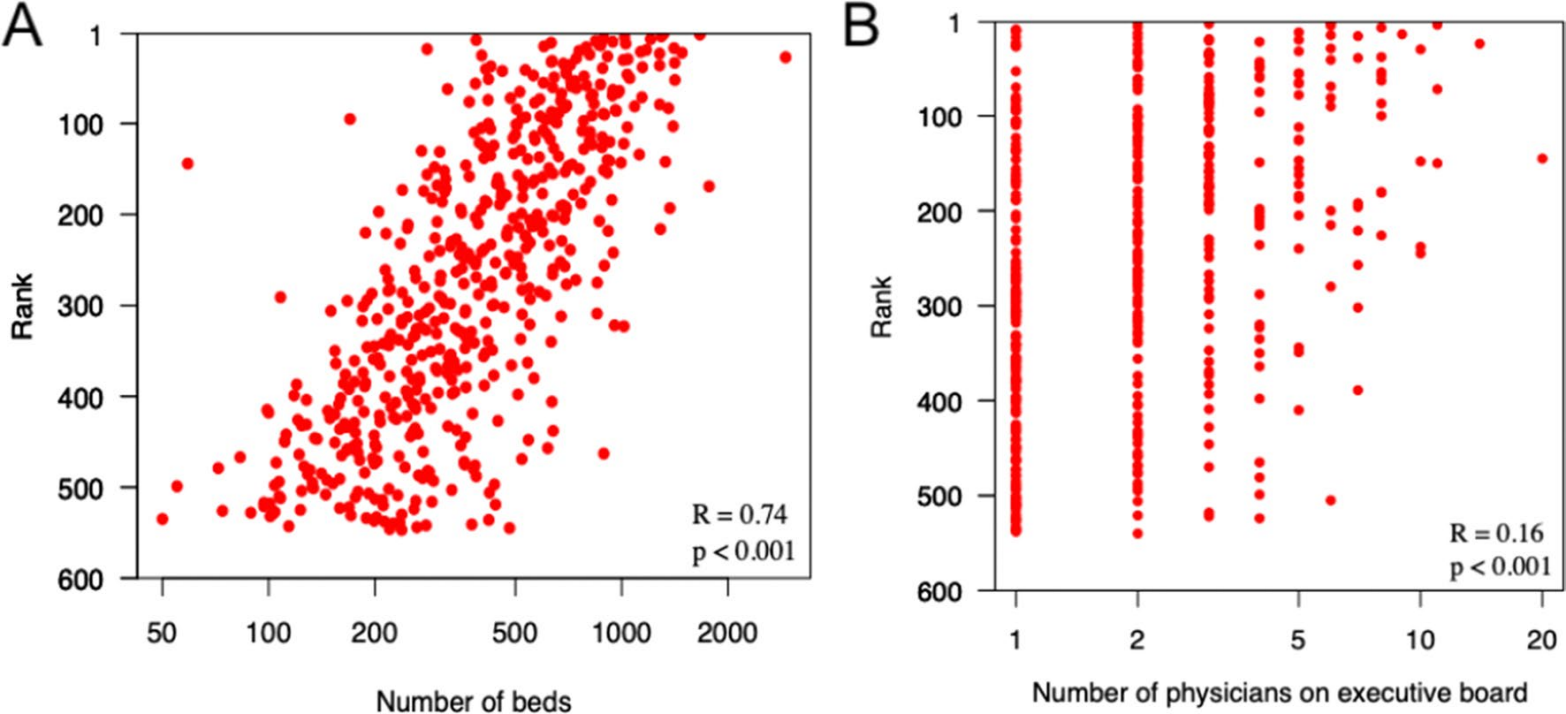
Correlation of **A** institution’s highest ranking hospital bed count and **B** number of physicians on the executive board with *US News and World Report* rank.

The median number of individuals on an executive board was 11 [8, 15], and the median number of physicians was 2 [1, 3]. Twenty-two percent (118/546) of institutions were led by a physician CEO (Table 2). The association between number of physician executive board members and institutional ranking (OR 1.05 (95% CI 0.99, 1.11) *P* = 0.111) suggested a potentially meaningful relationship despite not meeting criteria for statistical significance (Table 3). There was a significant positive association between institutional leadership under a physician CEO and higher *US News and World Report* ranking (Table 2, OR 1.59 (95% CI 1.27, 2.00) *P* < 0.001) (Fig. 1A). The likelihood ratio for physician vs non-physician CEO was significant for quartile 1 (Q1: LR = 11.25, *P* < 0.001), indicating concentration of physician CEOs in the top quartile (Other quartiles Q2: LR = 2.32, *P* = 0.128; Q3: LR = 0.13, *P* = 0.719; Q4: LR = 0.61, *P* = 0.433). The individual analysis showed a weak but statistically significant correlation (R = 0.16, *P* < 0.001) between the number of physician-executive board members and *US News and World Report* rank (Fig. 2B). We found no association between *US News and World Report* rank and executive board size.

**Table 3:** Multivariable Ordinal Regression of the Institutional Variables with *US News and World Report* Ranking as an Outcome Variable

Variable	Odds ratio (95% CI)	*P*-value
Physician CEO	1.59 (1.27, 2.00)	< 0.001*
Group practice	1.25 (1.01, 1.59)	0.042*
Executive board size (per person)	0.99 (0.97, 1.01)	0.478
Number of physicians on executive board (per person)	1.05 (0.99, 1.11)	0.111
Institution’s top ranking hospital bed count (per bed)	1.001 (1.0009, 1.0014)	< 0.001*
Institution’s bed count (per bed)	1.00003 (0.99996, 1.0001)	0.444
Number of hospitals in the institution (per hospital)	1.00 (0.99, 1.02)	0.483
For-profit	0.44 (0.24, 0.78)	0.005*

*p<0.05

### Organizational Structure and Incentive Alignment

Sixty-six percent (363/546) of institutions were group practices, and 5.1% (28/546) were for-profit institutions (Table 2). Group practice was positively associated with *US News and World Report* rank (OR 1.25 (95% CI 1.01, 1.59) *P* = 0.042), and institutions representing group practices ranked higher than non-group practices (Fig. 1B, Table 3). The likelihood ratio for group vs non-group practice was significant for quartile 4 (Q4: LR = 8.91, *P* = 0.002), indicating concentration of non-group practices in the bottom quartile (Other quartiles Q1: LR = 0.46, *P* = 0.498; Q2: LR = 1.03, *P* = 0.311; Q3: LR = 2.29, *P* = 0.130). There was an inverse relationship between for-profit status (OR 0.44 (95% CI 0.24, 0.78) *P* = 0.005) and institution ranking, with for-profit institutions ranking lower than not-for-profit (Fig. 1C, Table 3). The likelihood ratio for for-profit vs not-for-profit status was not significant for any of the quartiles; however, there was a trend towards significance for quartile 4 (Q4: LR = 3.05, *P* = 0.081), implying possible concentration of for-profits in the bottom quartile (Other quartiles Q1: LR = 1.51, *P* = 0.219; Q2: LR = 0.51, *P* = 0.474; Q3: LR = 0.95, *P* = 0.329).

### Institutional Size

The median number of beds in a health system was 1109 [609, 2227] (Table 2). The median number of beds in an institution’s top-ranked hospital (*n* = 546) was 379 [234, 625], and the median number of beds in a health system’s top-ranked hospital (*n* = 429) was 426 [266, 672]. There was a significant association between an institution’s top-ranked hospital bed count and *US News and World Report* ranking (OR 1.001 (95% CI 1.0009, 1.0014) *P* < 0.001) (Table 3). On an individual basis, there was a strong correlation between top-ranked hospital bed count and *US News and World Report* ranking (*R* = 0.74, *P* < 0.001) (Fig. 2A). We found no association between *US News and World Report* ranking and health system bed count or number of hospitals in a health system.

## DISCUSSION

When recognizably modern hospitals appeared in the late nineteenth century, the physician hierarchy was detached from the administrative leadership structure.^14^ Although this separation still exists, increasing integration is gradually obscuring the distinction between clinical leadership and organizational management.

Our findings show that certain features of healthcare organizational structures may carry predictive potential when applied to the *US News and World Report* rankings. Physician CEO was positively associated with *US News and World Report* rank. Furthermore, univariable analysis suggested a possible association between number of physician executive team members and US News ranking, a finding that raises important and complex questions about the importance of physician leadership and its relationship to healthcare quality as measured by *US News and World Report*. Previous studies investigating the frequently observed link between physician leadership and hospital performance have offered several explanations. In “Why the Best Hospitals Are Managed by Doctors,” Stoller et al. propose that physician-leaders who earn credibility in the clinical domain understand the needs and challenges of clinicians working underneath them and bring a patient-focused culture that non-clinician leaders are less adept to replicate.^15^ A different study showed that physician business leaders frequently bring a diverse collection of professional goals and compensation practices to their respective institutions that prioritize the patient experience, improve healthcare provider satisfaction, enhance institutional loyalty among workers, and strengthen employee trust in leadership.^16^ Another study found that among the *U.S. News and World Report* top 100 hospitals, there was a 25% difference in overall quality between physician-led hospitals and non-physician-led hospitals.^17^ These findings suggest that technical expertise at the executive level not only increases worker happiness and productivity but is likely among the most influential variables contributing to worker satisfaction.^18^

The results of the current analysis suggest an association between the flagship bed size and *US News and World Report* rank, an observation at least partially attributable to the well-established notion that providing highly specialized care is only financially viable within institutions of sufficient scale. Niche specialties such as cardiac oncology and transplant infectious disease can only exist at hospitals large enough to offset the costs associated with providing such specialized care.^19^ Although the odds ratio per unit change was smaller for bed count compared to other continuous variables, this could be attributed to the fact that unlike other continuous variables in the multivariable model that are less numerous, bed count has much wider range often exceeding 1000. In addition, our findings suggest that this observation may not be exclusively attributable to the influence of combining hospitals into larger health systems that inevitably have more beds. Furthermore, although consolidation may provide short-term financial gain by decreasing competition and centralizing resource allocation, no study has demonstrated that the financial costs of salaried, non-clinical administrative staffing are offset by superior patient care or efficiency.^20^

Our findings suggest that institutions utilizing group practice models achieve greater incentive alignment than their non-group practice competitors, a valuable element of the institutional architecture that fosters cooperation and collaboration between specialists. Although previous research in the social sciences has repeatedly demonstrated that cooperation and competition are not mutually exclusive, group practice models are more often associated with a collaborative work environment while overly competitive incentives can discourage mutually beneficial practices.^21^

Throughout the preceding decades, corporate influence and financial stakeholder involvement in United States healthcare has accelerated.^22^ Advocates of the for-profit model emphasize the positive impact of market pressures on innovation and the value of competition for maximizing the efficiency of healthcare delivery and reducing costs.^23,24^ Critics argue that greater involvement of profit-seeking financial institutions and corporate actors negatively impacts hospitals in a variety of essential ways. Quality improvement studies have repeatedly demonstrated a strong association between private equity ownership and the erosion of the patient-physician relationship and increased likelihood of physician burnout and moral injury.^25,26^ This reduces the relationship to transactional rather than focusing on cultivating trusting and transparent relationships with patients.^27^ Our finding that for-profit status was associated with lower *US News and World Report* ranking is aligned with these reports, and although for-profit institutions comprise a minority of all health systems, they represent a sizeable portion with respect to hospital number and bed count and should not be overlooked.

Our study contains several limitations that are important to acknowledge. First, our analysis was limited by the number of available predictor variables. It is possible that our findings could have revealed different associations, perhaps even supporting alternate conclusions, had additional predictor variables been available and included in the analysis. Our approach was to analyze all available variables simultaneously in a multivariable model, and variables found to be significantly associated with the outcome in the multivariable model were analyzed individually in univariable analyses. Adherence to this approach limited our capability to identify relationships within data-scarce territories. For example, our study could not account for heterogeneity between for-profit and not-for-profit sub-organizations of a non-profit parent because the data volume and granularity required to perform such an analysis is currently lacking. Furthermore, we sought to maintain an accurate and consistent definition of “group practice” without conflating the relationship between physicians practicing within a single group practice and other relationships that exist between administrative and clinical personnel. In pursuit of this aim, our study defined “group practice” using the AHRQ definition as a group of physicians that offer services under a shared entity, and this definition would have excluded non-integrated groups of physicians who provide patient care as a collective entity which could impact our results and conclusions. Lastly, demographic leadership variables such as age, gender, and medical specialty were not amenable to systematic collection and were not pursued in this analysis.

Our investigation focused on a single ranking system, increasing the study susceptibility to inaccuracies within the *US News and World Report* institutional practices and vulnerability to the influence of biases within their methodological processes. The limitations of *US News and World Report* hospital ranking metrics are well-documented, and although institution rank has never been demonstrated to correlate with patient outcomes, concluding that because hospital ranking is correlated with volume which has repeatedly been linked to patient outcomes suggests the presence of a connection between patient outcomes and *US News and World Report* ranking could be misguided. Furthermore, reputational score was part of the *US News and World Report* ranking and, as such, a subjective metric made its way into a numeric score. Therefore, we recognize that *US News and World Report* hospital rankings represent imperfect surrogates for health system performance and the overall ranking system remains a controversial measure of institution quality which potentially lessens the value of our conclusions. Such observations underscore the importance of exercising caution when interpreting and extrapolating data.

*US News and World Report* relies on data provided by institutions and third-party entities, so our study would be negatively impacted by any shortcomings or substantial omissions in data reporting, as well as the possibility that the strength of association between hospital bed count and *US News and World Report* rank is impacted by their methodologic processes. Lastly, because there is no simple binary classifier for a coarse-grained description of an institution’s academic status, exploring the impact of academic affiliation on health outcomes quality was determined to be beyond the scope of analysis for this study.

In summary, our study highlights an array of factors that are associated with superior institutional performance as measured by the annual *US News and World Report* rankings. It is likely that physician leadership, incentive practices, flagship hospital size, and not-for-profit status all contribute to organizational performance in complex ways, of which some are better understood than others. Nevertheless, we hope that our findings may be used to inform policymaking at the state or national governmental level while acknowledging that if we strive to deliver healthcare that is effective, affordable, and equitable, our leadership models and governance practices must be evidence-based, and our healthcare institutions and decision-makers within them must reflect the values and priorities of the patients they serve.

## Supplementary Information

Below is the link to the electronic supplementary material.

Supplementary file1 (DOCX 16 KB)

## Data Availability

The data supporting the findings of this study can be made available upon request. Requests for access can be directed to the corresponding author.
