# Supplementary material for: Leadership Practices, Organization Structure, and Other Factors Associated with Higher 2022–2023 US News and World Report Ranking
Source: J Gen Intern Med. 2024 Oct 2;40(16):3909–15. doi: 10.1007/s11606-024-09028-7 (PMC12686240; doi:10.1007/s11606-024-09028-7)
Supplement: Supplementary file 1 — Supplementary file1 (DOCX 16 KB) [file 11606_2024_9028_MOESM1_ESM.docx]

**Supplementary Table 1:** *US News* honor roll hospitals (2022-2023)

| **Rank** | **Hospital** | **Location** |
| --- | --- | --- |
| 1 | Mayo Clinic | Rochester, MN |
| 2 | Cedars-Sinai Medical Center | Los Angeles, CA |
| 3 | NYU Langone Hospitals | New York, NY |
| 4 | Cleveland Clinic | Cleveland, OH |
| 5 | Johns Hopkins Hospital | Baltimore, MD |
| 5 | UCLA Medical Center | Los Angeles, CA |
| 7 | New York-Presbyterian Hospital-Columbia and Cornell | New York, NY |
| 8 | Massachusetts General Hospital | Boston, MA |
| 9 | Northwestern Memorial Hospital | Chicago, IL |
| 10 | Stanford Health Care-Stanford Hospital | Stanford, CA |
| 11 | Barnes-Jewish Hospital | St. Louis, MO |
| 12 | UCSF Health-UCSF Medical Center | San Francisco, CA |
| 13 | Hospitals of the University of Pennsylvania-Penn Presbyterian | Philadelphia, PA |
| 14 | Brigham and Women's Hospital | Boston, MA |
| 15 | Houston Methodist Hospital | Houston, TX |
| 16 | Mount Sinai Hospital | New York, NY |
| 17 | University of Michigan Health-Ann Arbor | Ann Arbor, MI |
| 18 | Mayo Clinic-Phoenix | Phoenix, AZ |
| 19 | Vanderbilt University Medical Center | Nashville, TN |
| 20 | Rush University Medical Center | Chicago, IL |

**Supplementary Table 2**: Top 20 health systems based on pooled *US News* scores

| **Rank** | **Health System** | **Location** |
| --- | --- | --- |
| 1 | Mayo Clinic | Rochester, MN |
| 2 | NYU Langone Health | New York, NY |
| 3 | NewYork-Presbyterian | New York, NY |
| 4 | Cleveland Clinic Health System | Cleveland, OH |
| 5 | Johns Hopkins Health System | Baltimore, MD |
| 6 | Cedars-Sinai Health System | Los Angeles, CA |
| 7 | Mount Sinai Health System | New York, NY |
| 8 | University of Pennsylvania Health System | Philadelphia, PA |
| 9 | Houston Methodist | Houston, TX |
| 10 | Northwestern Memorial HealthCare | Chicago, IL |
| 11 | Rush University System for Health | Chicago, IL |
| 12 | Michigan Medicine | Ann Arbor, MI |
| 13 | UCSF Health | San Francisco, CA |
| 14 | Mass General Brigham | Boston, MA |
| 15 | Stanford Health Care | Stanford, CA |
| 16 | UTSW Health System | Dallas, TX |
| 17 | BJC HealthCare | St. Louis, MO |
| 18 | UCLA Health | Los Angeles, CA |
| 19 | Vanderbilt Health | Nashville, TN |
| 20 | UC San Diego Health | La Jolla, CA |
